# Conformational Dynamics and Antigenicity in the Disordered Malaria Antigen Merozoite Surface Protein 2

**DOI:** 10.1371/journal.pone.0119899

**Published:** 2015-03-05

**Authors:** Christopher A. MacRaild, Milan Zachrdla, Dean Andrew, Bankala Krishnarjuna, Jiří Nováček, Lukáš Žídek, Vladimír Sklenář, Jack S. Richards, James G. Beeson, Robin F. Anders, Raymond S. Norton

**Affiliations:** 1 Medicinal Chemistry, Monash Institute of Pharmaceutical Sciences, Monash University, 381 Royal Parade, Parkville, 3052, Australia; 2 NCBR, Faculty of Science, Masaryk University, Kamenice 5, 62500, Brno, Czech Republic; 3 CEITEC, Masaryk University, Kamenice 5, 62500, Brno, Czech Republic; 4 Centre for Biomedical Research, Burnet Institute, Melbourne, Victoria, 3004, Australia; 5 Department of Biochemistry, La Trobe University, Victoria, 3086, Australia; Ehime University, JAPAN

## Abstract

Merozoite surface protein 2 (MSP2) of *Plasmodium falciparum* is an abundant, intrinsically disordered protein that is GPI-anchored to the surface of the invasive blood stage of the malaria parasite. Recombinant MSP2 has been trialled as a component of a malaria vaccine, and is one of several disordered proteins that are candidates for inclusion in vaccines for malaria and other diseases. Nonetheless, little is known about the implications of protein disorder for the development of an effective antibody response. We have therefore undertaken a detailed analysis of the conformational dynamics of the two allelic forms of MSP2 (3D7 and FC27) using NMR spectroscopy. Chemical shifts and NMR relaxation data indicate that conformational and dynamic properties of the N- and C-terminal conserved regions in the two forms of MSP2 are essentially identical, but significant variation exists between and within the central variable regions. We observe a strong relationship between the conformational dynamics and the antigenicity of MSP2, as assessed with antisera to recombinant MSP2. Regions of increased conformational order in MSP2, including those in the conserved regions, are more strongly antigenic, while the most flexible regions are minimally antigenic. This suggests that modifications that increase conformational order may offer a means to tune the antigenicity of MSP2 and other disordered antigens, with implications for vaccine design.

## Introduction

Recent decades have seen an increasing recognition that many proteins naturally lack a defined folded state, and that their function depends instead on conformational disorder [1,2]. Such proteins are termed intrinsically unstructured or disordered proteins, and are found across all of biology. In particular, intrinsically disordered proteins are abundant in a range of pathogenic organisms. The proteomes of some viruses are predicted to be almost entirely disordered [3], and several parasite species also have an unusually high proportion of disordered proteins [4]. Nonetheless, the implications of protein disorder for immune recognition by B cells and antibodies have received remarkably little attention [5]. On the one hand, it has been suggested that intrinsically disordered proteins generally elicit weak immune responses or are even completely non-immunogenic [6]. It has been observed that functionally important sites on protein antigens are highly flexible, or are surrounded by flexible loops. This flexibility is proposed in some instances to serve as a means of immune evasion [7]. In sharp contrast to this view, however, it has been suggested that disordered antigens are in some contexts immunodominant [8], but that they fail to contribute to an effective immune response. Thus, they are believed to function for some pathogens as a smoke screen, diverting the immune system from targets with greater protective potential [9]. Nonetheless, numerous B-cell epitopes have been characterised in disordered proteins, and many of these appear to contribute to functional immune responses and therefore represent potential vaccine candidates [5,10–17]. For example, the protective effects of RTS,S, the most advanced malaria vaccine in clinical development, appear to be mediated by antibodies to the disordered repeats of the circumsporozoite protein [15,18].

In order to better understand the effects of conformational disorder on the immune response, and to contribute to the development of a malaria vaccine, we have investigated merozoite surface protein 2 (MSP2). MSP2 is an abundant component of the surface coat of the *Plasmodium falciparum* merozoite, the form of the parasite that invades red blood cells during the blood-stage of infection, which is responsible for symptomatic and severe malaria. Although the specific function of MSP2 has not been defined, it appears to play an essential role in blood-stage replication; it is retained on the merozoite surface during invasion and then degraded soon after invasion is complete [19]. An extensive body of evidence implicates MSP2 as a potential target of protective immunity against *P*. *falciparum* infection [20–26]. Antibodies to MSP2 have been associated with protection from malaria in prospective longitudinal studies [27–29] and MSP2 antibodies promote opsonic phagocytosis of merozoites and antibody-dependent cellular inhibition of blood-stage replication [26,30,31].

**Fig 1:**
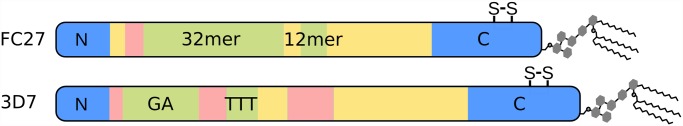
Schematic depiction of the primary structure of the two allelic families of MSP2. The conserved N- and C-terminal regions of MSP2 are in blue, while the allele-specific central region is composed of polymorphic repeats (green) and non-repetitive sequences (pink) as well as dimorphic regions (yellow) that differ between the allelic families but are conserved within them. The position of the conserved disulfide bond in the C-terminal regions is indicated.

MSP2 is highly polymorphic, with conserved N- and C-terminal domains flanking a central variable region, which contains tandemly arrayed repetitive sequences [32,33]. All MSP2 alleles have been categorized into two families typified by the 3D7 and FC27 alleles, respectively, because of differences in the repeats and flanking variable sequences (Fig. 1) [32,34,35]. Indeed, the sequence variability within each allelic family is limited to the repeat regions and to a few localised regions of heterogeneity within the regions flanking the repeats (green and pink in Fig. 1).

MSP2 is a candidate for inclusion in a malaria vaccine [36], and the 3D7 allele of MSP2 was a component of a subunit vaccine that significantly reduced parasite densities in a clinical trial in Papua New Guinea [25]. This vaccine showed protective efficacy against infections with parasites expressing the vaccine-like 3D7-type MSP2 sequence, indicating that vaccine efficacy was mediated by strain-specific responses to MSP2 [23]. Efforts to elicit protective antibodies against the conserved regions of MSP2 are complicated by the observation that anti-MSP2 antibodies induced by infection with *P*. *falciparum* are largely directed against epitopes in the central variable region of the molecule [37,38], and that many conserved-region epitopes are cryptic on the parasite surface [10]. As such, the generation of a broadly effective MSP2-based vaccine is likely to require fine control of the specificity of the induced immune response. In this context, we have undertaken a detailed study of the conformational dynamics of MSP2, with the goal of establishing the extent to which these properties might contribute to the observed patterns of antigenicity and immunogenicity against MSP2, and the extent to which they might be exploited to fine-tune the specificity of the antibody response against MSP2.

## Methods

### Materials

Untagged full-length FC27 MSP2 was expressed and purified using a strategy specific for recombinantly expressed disordered proteins, as described previously [39]. A synthetic gene encoding 3D7 MSP2, codon optimised for expression in *Escherichia coli* (Genescript), was cloned into pET32a (Novagen) using KpnI and NcoI. The resulting construct contains an N-terminal thioredoxin (Trx) and His_6_-tag for affinity purification. Bacterial cell pellets were lysed by heating, as for FC27 MSP2 [39]. The expressed fusion protein was isolated on a HisTrapFF affinity column (GE Healthcare), eluted with imidazole and cleaved with 1% (w/w) TEV protease. The released Trx-tag and any uncleaved fusion protein were subsequently removed by a second passage through the His-trap column. Final purification of 3D7 MSP2 was by HPLC, using a C18 column (0.9 x 25 cm, Zorbax) and a linear acetonitrile gradient in 0.1% TFA. Isotopically enriched 3D7 and FC27 MSP2 for NMR studies was prepared by growing expression cultures in M9 minimal medium, with 1 g/L ^15^N ammonium chloride and/or 4 g/L ^13^C glucose as the sole nitrogen and carbon sources, respectively. The final recombinant 3D7 MSP2 has an N-terminal Gly derived from the TEV cleavage site whereas recombinant FC27 MSP2 has an N-terminal Met derived from the start codon.

### NMR spectroscopy

NMR samples contained 0.4 mM 3D7 or FC27 MSP2 in 50 mM sodium acetate, pH 4.5, with 7% ^2^H_2_O included for the spectrometer lock. All of the data used for resonance assignments were acquired on a 700 MHz Bruker Avance III spectrometer equipped with the ^1^H/^13^C/^15^N TXO cryogenic probehead with z-axis gradients at 25°C. The HNCO spectrum was acquired with spectral widths set to 9800 (aq) x 2500 (^15^N) x 2000 (^13^C’) Hz, and with maximal evolution times of 80 ms (^13^C’) and 80 ms (^15^N) in the indirectly detected dimensions. The inter-scan delay was set to 1.1 s, and 4 transients per free induction decay (FID) were cumulated. The overall number of 2048 complex points was acquired in the acquisition dimension, whereas 600 hypercomplex points were randomly distributed over the indirectly-detected dimensions. The experiment was acquired in 3.5 h, which represents 1.9% of the time needed for a conventional experiment with similar settings. The 5D HN(CA)CONH experiment was acquired with the spectral widths set to 9800 (aq) x 2500 (^15^N) x 2000 (^13^C’) x 2800 (^15^N) x 8000 (^1^H) Hz [40]. The maximal acquisition times were adjusted to 15 ms for the ^1^H indirectly-detected dimension, to 27 ms and 40 ms for ^15^N dimensions, and to 30 ms for the ^13^C’ dimension. The experiment was acquired with 2048 complex points in the acquisition dimension and 1750 hypercomplex points were randomly distributed over the indirectly detected dimensions. The inter-scan delay was set to 1.25 s and 4 transients per FID were collected. The experimental time of 46 h represents 0.0036% of the time needed for a similar experiment using conventional settings. The 5D HabCabCONH experiment was acquired with spectral widths set to 9800 (aq) x 2500 (^15^N) x 2000 (^13^C’) x 10000 (^13^C^aliph^) x 5000 (^1^H^aliph^) [40]. The maximal evolution times were set to 12 ms for ^1^H^aliph^, 6.5 ms for ^13^C^aliph^, 30 ms for ^13^C’, and 22 ms for ^15^N indirect dimensions. The total number of 1536 complex points was measured in the directly-detected dimension, and 1750 hypercomplex points were randomly distributed in the indirectly-detected dimensions. The experiment was acquired with 4 transients per collected FID and an interscan delay of 1.25 s. The overall experimental time of 46 h represents 0.008% of the time needed for acquisition of the conventional experiment providing similar resolution.

NMR relaxation experiments were performed on a 600 MHz Bruker Avance III NMR spectrometer equipped with a QCI-P cryogenic probehead with z-axis gradients at 25°C. Temperature was calibrated according to the chemical shift differences of pure methanol peaks. Spectral widths were set to 8370 (aq) x 1428 (^15^N) Hz. The overall number of 2048 complex points was acquired in the acquisition dimension and 400 complex points were acquired in the indirect dimension for auto-relaxation rates R_1_, R_2_, cross-correlated relaxation rates Γ_x_, Γ_z_ and steady state ^15^N-^1^H nuclear Overhauser effect (NOE) [41]. Standard experiments were used for the measurement of R_1_ (relaxation delays 11.2, 56, 134.4, 235.2, 380.8, 560, 896*, 1344, 1848, and 2352 ms) and R_2_ (relaxation delays 0, 14.4, 28.8*, 43.2, 57.6, 72*, 86.4, 115.2, and 144 ms) [42]. Asterisks denote spectra recorded twice in order to estimate experimental error. Experiments based on symmetrical reconversion were performed for determination of transverse cross-correlated relaxation rates Γ_x_ (relaxation delays 30, 50, and 70 ms) and longitudinal cross-correlated relaxation rates Γ_z_ (relaxation delays 100, 150, 200, and 250 ms) [43,44].

### Antigenicity

Antigenicity across the MSP2 sequence was determined using sera from mice and rabbits immunised with full-length recombinant 3D7 or FC27 MSP2 (Genebank JN248383 and JN248384). Both proteins were expressed in *E*. *coli* with C-terminal His_6_ tags and purified by metal-chelating, anion-exchange and reverse-phase chromatography [31]. Animals were immunised with the recombinant MSP2 formulated in Montanide ISA720. Mice (C57Bl/6) were immunised with 10 μg subcutaneously and rabbits were immunised with 100 μg intramuscularly on two occasions with a four-week interval between immunisations. Serum samples used in antigenic analyses were obtained from blood samples collected two weeks after the second immunization. Immunisations were approved by the La Trobe University Animal Ethics Committee and were conducted in accord with the policies of the National Health and Medical Research Council, Australia. Reactivity to a panel of 13-residue biotinylated peptides covering the sequence of both antigens with an 8-residue overlap, was measured by ELISA, as described previously [10,19]. The panel contains one copy of the first three peptides common to both 3D7 and FC27 MSP2, but because the central variable regions of 3D7 and FC27 MSP2 are different lengths, the two peptide sets (3D7 and FC27) extended through the conserved C-terminal region to give two sets of peptides covering the same sequence but out of frame with respect to each other, as described previously [10]. Sixteen sera (four per condition) were analysed in triplicate, with all sera tested at 1:1000 dilution and secondary antibody diluted 1:2000. Responses from unimmunised animals were also measured, and a background signal three standard deviations greater than the mean of these responses was subtracted from all results. Agreement between animals was assessed using Pearson’s correlation coefficients for pairwise comparisons of mean ELISA results for each animal. Permutation tests were used to estimate two-tailed p-values. Within each condition, each residue in MSP2 was accorded the average response of all peptides in which that residue is represented and the resulting antigenicity profiles were normalised.

## Results

### Backbone resonance assignments for 3D7 MSP2

The assignment of observed spectral frequencies (chemical shifts) in an NMR spectrum to specific atoms in the protein is a prerequisite for detailed structural analysis by NMR, allowing measured spectral parameters to be ascribed to specific structural features. We have previously determined near-complete backbone assignments for FC27 MSP2, but expression yields for 3D7 MSP2 were insufficient to permit the detailed analysis of that allelic form [39]. Here, we employ a new expression system, based on a thioredoxin fusion strategy, that yields ~ 10 mg 3D7 MSP2 per litre of culture medium. An assignment strategy tailored to repetitive disordered proteins and exploiting two 5D experiments, HN(CA)CONH and HabCabCONH, was employed to assign the resonance frequencies of 3D7 MSP2 [45]. All the non-proline residues were successfully assigned, although residues 37–58, within the GGSA repeats, show degenerate backbone chemical shifts, as do residues 77, 78, and 84–87 within the TTT repeats.

**Fig 2:**
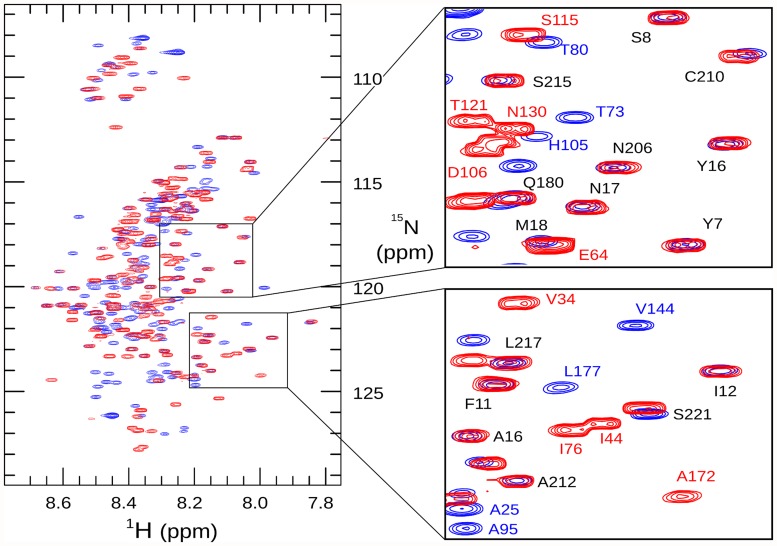
^1^H,^15^N heteronuclear single-quantum correlation spectra of 3D7 (blue) and FC27 (red) MSP2. Assigned peaks are labelled in the expanded regions, highlighting the similarity of chemical shift for these residues in the conserved N- and C-terminal regions (labelled in black type, FC27 numbering) of the two allelic forms of MSP2.

**Fig 3:**
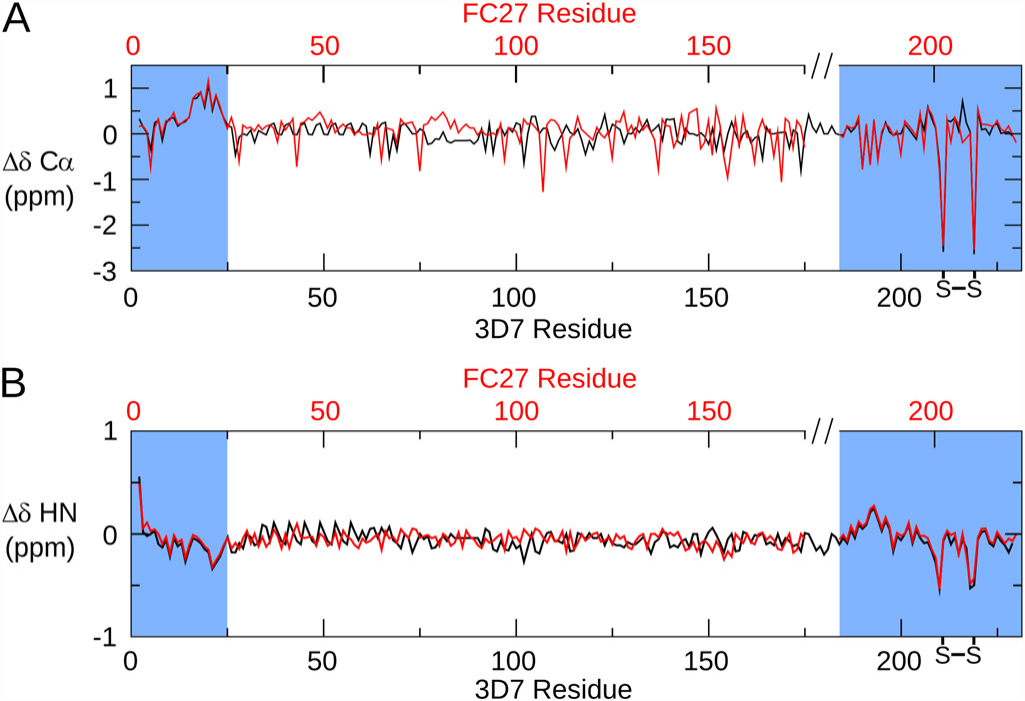
Secondary chemical shifts of MSP2. The difference between the observed Cα (A) and HN (B) chemical shifts and those predicted for a disordered protein by the method of Tamiola et al. [50] is plotted for 3D7 (black) and FC27 (red) MSP2. The data for FC27 are plotted on a broken axis (top) in order to correctly align the conserved regions.

The amide chemical shifts of 3D7 MSP2 show minimal dispersion (Fig. 2), and backbone shifts are close to those expected for a disordered protein (Fig. 3). This demonstrates that 3D7 MSP2, like FC27, is extensively disordered, consistent with our previous analyses [39,46]. Comparison of the backbone chemical shifts of the FC27 and 3D7 forms of MSP2 reveals almost perfect correspondence between the shifts of the conserved N and C-terminal regions, indicating that the conformational propensities of these regions are identical in the two allelic forms (Figs. 2 and 3). In particular, slightly elevated Cα secondary chemical shifts are seen in the N-terminal region of both MSP2 forms, indicating a weak preference for helical conformation in this region (Fig. 3) [39,47,48]. Cβ chemical shifts of the two Cys residues confirm the presence of the single disulfide in MSP2 [49].

### Conformational dynamics probed by ^15^N relaxation

**Fig 4:**
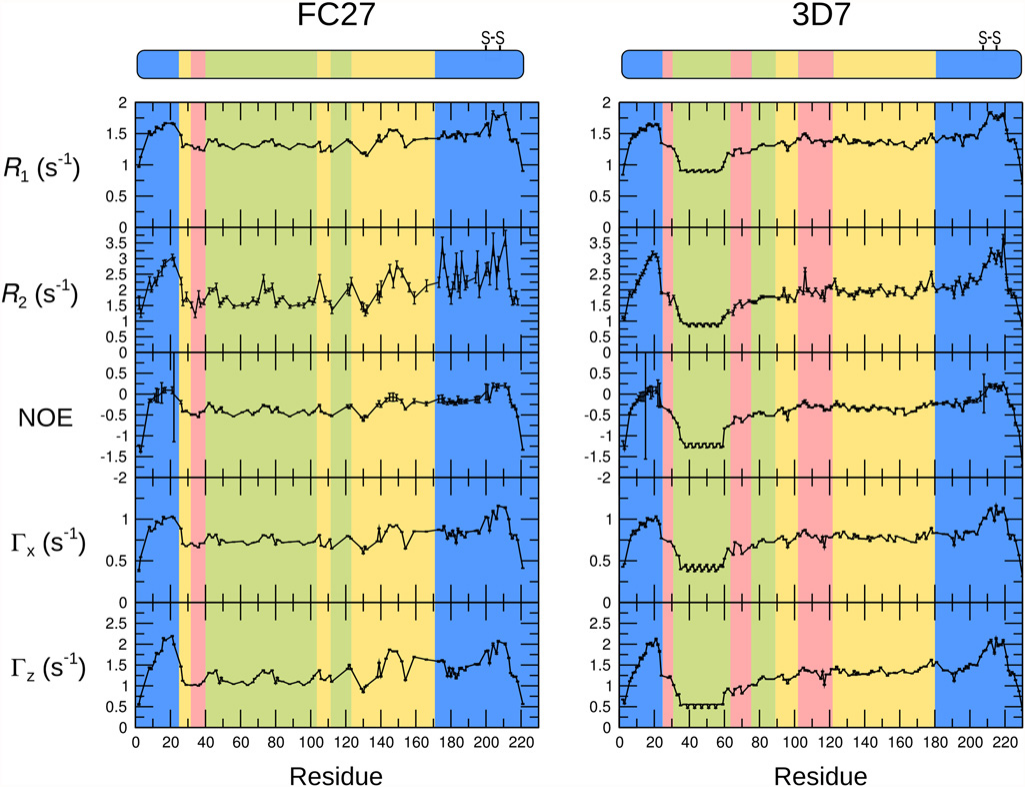
The ^15^N auto-relaxation rates *R*_1_, *R*_2_, cross-correlated relaxation rates Γ_x_, Γ_z_, and steady-state ^15^N-^1^H NOE for FC27 (left) and 3D7 (right) MSP2. The sequence regions of MSP2 are colour-coded as in Fig. 1.

**Fig 5:**
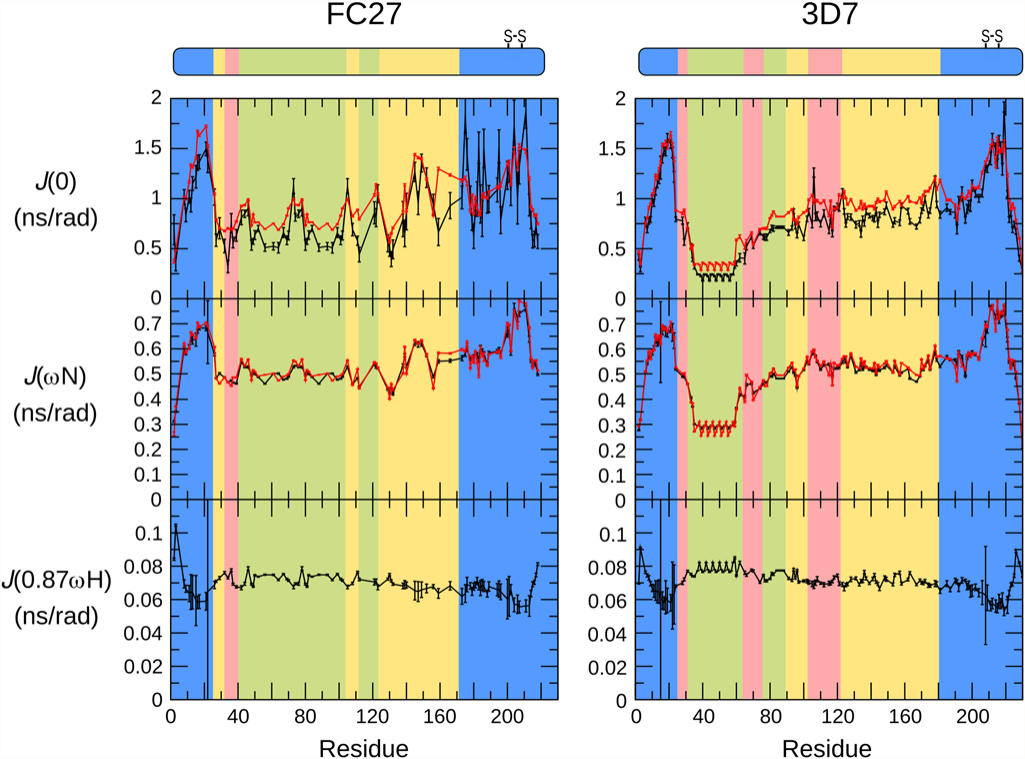
Values of the ^15^N spectral density functions for FC27 (left) and 3D7 (right) MSP2, as determined from reduced spectral density mapping from the auto-relaxation rates and steady-state ^15^N-^1^H NOE (black) and cross-correlated relaxation rates (red). The sequence regions of MSP2 are colour-coded as in Fig. 1.

NMR relaxation rates are sensitive to fast conformational dynamics [51], and as such are valuable probes of the extent of disorder in unstructured proteins [52]. We have measured relaxation rates of the backbone amides of MSP2 to determine conformational dynamics at ps-ns timescales and at single-residue resolution (Fig. 4). Values of the spectral density function *J*(ω) at zero frequency and at the ^15^N and ^1^H Larmor frequencies were calculated from ^15^N relaxation rates (Fig. 5) [53]. These values represent the direct link between the experimental data and the conformational dynamics of the protein, with a larger value of *J*(ω) indicating a larger contribution to relaxation from dynamic processes with frequency ω. In order to identify residues exhibiting dynamics at μs-ms timescales, the *J*(0) and *J*(ω_N_) values were calculated from both auto-correlated (*R*_1_, *R*_2_, steady-state [^1^H]-^15^N NOE) and cross-correlated (Γ_z_, Γ_x_) relaxation data [54].

The results of the relaxation measurements are consistent with those expected for a disordered protein; relaxation rates are uniformly low, while the steady-state [^1^H]-^15^N NOE is generally negative. These results reflect conformational dynamics dominated by local processes on timescales shorter than 1 ns. In contrast, the relaxation of a conventionally structured protein is dominated by overall rotational diffusion (on a timescale >10 ns, for a protein the size of MSP2), with relatively small contributions from faster local processes. For FC27 MSP2 we observe relaxation properties that are in excellent agreement with those we reported previously under more acidic conditions [39]. Under both conditions, we observe more rapid relaxation and smaller magnitude (and in some cases small positive) ^1^H-^15^N NOEs, consistent with a degree of conformational constraint, in the following three distinct regions: throughout the conserved N-terminus, in part of the C-terminal region coincident with the single disulfide bond in MSP2, and in part of the FC27-specific dimorphic region, between residues 140 and 150 (Figs. 4 and 5). It should be stressed that, although these regions are more ordered than the rest of MSP2, they do not represent regions of folded regular structure as both their relaxation properties and chemical shifts are indicative of significant residual disorder, well beyond that observed in conventional structured proteins. Rather, the flexibility of these regions is weakly constrained by transient helical structure in the N-terminal region, and by the disulfide in the C-terminal region. For a few residues in the C-terminal conserved region, values of *J*(0) calculated from auto-correlated relaxation data are larger than those obtained from the cross-correlated relaxation (Fig. 5). This is suggestive of exchange contributions to the measured *R*_2_ relaxation rates for these residues, and may imply the existence of a weakly populated meta-stable conformational state with a lifetime in the μs-ms range [55]. The repeat regions of FC27 MSP2 show somewhat variable dynamic properties, with elevated values of *J*(ω_H_), indicating more extensive sub-ns dynamics than observed in the rest of the dimorphic and C-terminal regions, but with significant variation in the lower-frequency spectral densities across the 32-residue repeat (Fig. 5).

The variable region of 3D7 MSP2 shows greater diversity in its dynamic properties, as reported by relaxation measurements. The largest region of polymorphism in 3D7 MSP2, the GGSA repeats, is exceptionally flexible, with relaxation properties indistinguishable from the extreme termini of the protein (Fig. 5; residues 32–63). Presumably this flexibility is a consequence of the uniformly small side chains in this region, and the correspondingly small steric barriers to backbone reorganisation. It is noteworthy that, despite the high levels of polymorphism, this region is consistently rich in small residues, with Gly, Ser and Ala representing over 90% of residues seen in this region across all 3D7 MSP2 alleles characterised. In contrast, the next largest region of polymorphism, residues 103–122 appears relatively ordered, to essentially the same degree as the more ordered region in the FC27 dimorphic domain, residues 140–150. Likewise, the degree of order in the 3D7 dimorphic region (residues 123–180; yellow in Figs. 4 and 5) is comparable to the remainder of the FC27 dimorphic region, and the 3D7 TTT repeats are comparable to the FC27 32- and 12-residue repeats.

In contrast to the variable regions, the dynamic properties of the conserved regions of MSP2 are indistinguishable in the two allelic forms at ps-ns timescales, as indicated by identical relaxation rates, with the regions of reduced flexibility within the N- and C-terminal regions being the most ordered regions in both alleles (Fig. 3). Together with the perfect correspondence of backbone chemical shifts, this agreement indicates that the ensembles of rapidly inter-converting conformational states sampled by these regions are identical in FC27 and 3D7 MSP2. The intervening variable regions exert no perceptible influence on these properties. More generally, this suggests that the conformational properties of MSP2 are entirely locally determined. This inference is consistent with our observation that each member of a large panel of monoclonal antibodies recognises a simple linear epitope [10].

For 3D7 MSP2, the *J*(0) values calculated from auto-correlated relaxation data are never significantly larger than those obtained from cross-correlated relaxation (Fig. 5), suggesting that the μs-ms dynamics that was inferred for FC27 MSP2 C-terminal conserved region are absent in this region of 3D7 MSP2. This suggests that the variable region may influence the population or lifetime of meta-stable conformations in the conserved C-terminal domain. Remarkably, this appears to occur without affecting the ps-ns dynamics, or the overall conformational preferences (as reported by chemical shift) of either region.

### Antigenicity is correlated with local dynamics

**Fig 6:**
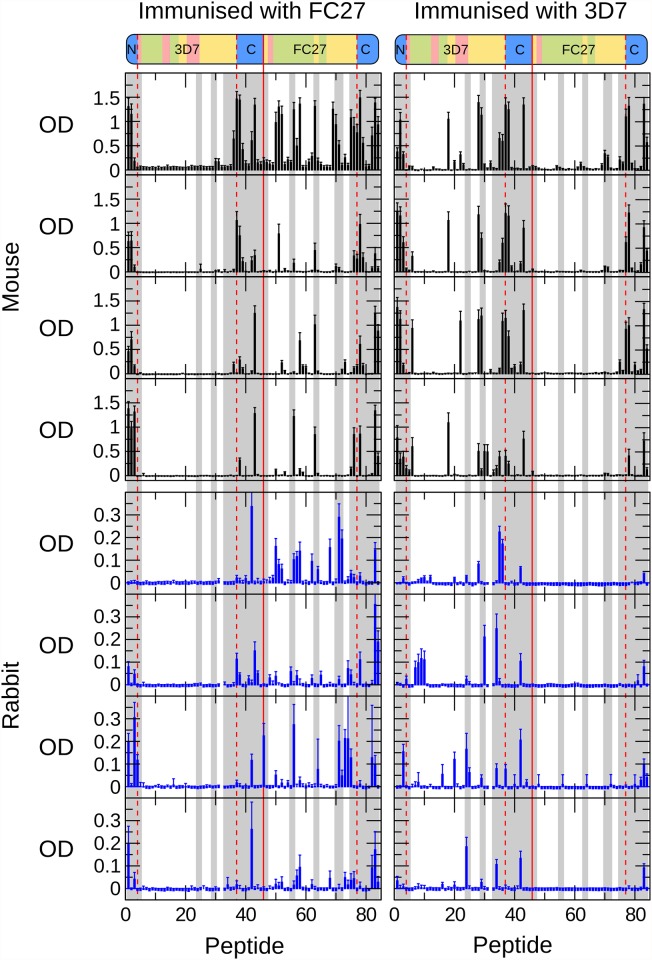
Antigenic profile of MSP2 mapped by ELISA. Four mice (top panels, black bars) and four rabbits (bottom panels, blue bars) were immunised with either FC27 (left panels) or 3D7 (right panels) MSP2. Individual immune sera were tested against a single panel of overlapping peptides covering the sequences of both 3D7 MSP2 (peptides 1–45) and FC27 MSP2 (peptides 46–84), as shown schematically above. The conserved N terminal (peptides 1–3) and C terminal (peptides 37–45 and 77–84) regions are common to both 3D7 and FC27 MSP2 and are delineated with dashed red lines. Peptides showing greater than the median level of conformational restriction are shaded grey (Table 1). Mean optical density from triplicate assays is plotted for each serum, corrected for the response from unimmunised control sera. Error bars are one standard deviation.

**Fig 7:**
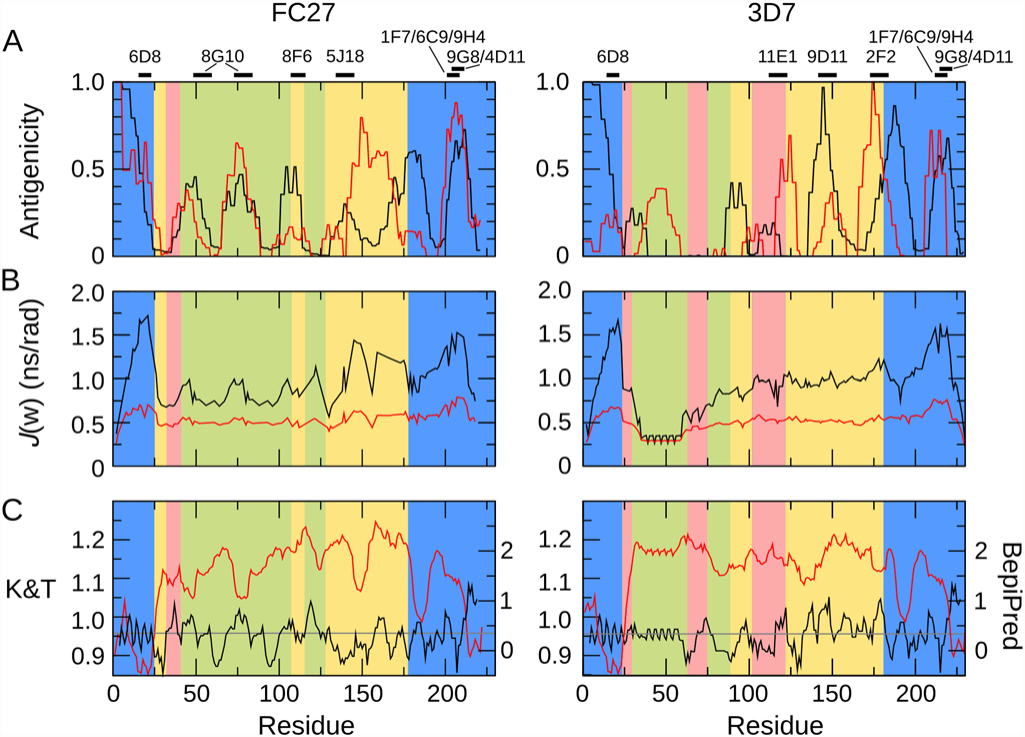
Comparison of experimental patterns of antigenicity, predicted antigenicity, and conformational dynamics, for FC27 (left) and 3D7 (right) MSP2. A. Antigenicity profiles of MSP2 inferred from experimental immunisation of mice (black) and rabbits (red) are plotted against the sequence. Black bars (top) denote the location of epitopes of a panel of monoclonal antibodies to MSP2. B. Conformational flexibility of MSP2 as measured by the spectral density functions derived from the ^15^N relaxation data. Spectral density functions are plotted at zero frequency (black line) and at the ^15^N Larmor frequency (red line). C. Antigenicity of MSP2 as predicated using BepiPred [60] (red, right axis) and the method of Kolaskar and Tongaonkar [61] (black, left axis). The threshold for epitope prediction for both methods is denoted by the grey line.

Potential correlations between the conformational dynamics characterised above and the antigenicity of MSP2 have been investigated by examining the patterns of local antigenicity in sera of mice and rabbits immunised with recombinant 3D7 and FC27 MSP2. The reactivity of MSP2 antisera to an array of overlapping peptides covering the entire sequences of 3D7 and FC27 MSP2 was measured by ELISA [10,19]. To enable direct comparison between these results and our NMR measurements, which are resolved at the level of individual residues, we adopted a scoring scheme in which each residue was scored according to the average reactivity of each of the peptides in which that residue was represented. There was good agreement across the individual mice immunised against each antigen. Pairwise comparisons of ELISA results from individual mice immunised with 3D7 MSP2 yielded average correlation coefficients of 0.7 ± 0.1 and for mice immunised with FC27 MSP2 the average correlation was 0.6 ± 0.1 (Pearson’s r; p<10^-5^ for all comparisons). For rabbits, there is substantially greater variation between individuals, with correlation coefficients of 0.3 ± 0.2 for each antigen (p<0.05 for seven of 12 comparisons). Nonetheless there was reasonable qualitative agreement across all antigenicity profiles for each antigen, with most regions identified to be antigenic in mice also antigenic in at least one rabbit, and vice versa (Fig. 6). The following analysis therefore considers a single average profile for each antigen in each species (Fig. 7A).

**Table 1:** Conformationally constrained peptides are more antigenic.

			No. animals responding^b^			No. peptides with > 2 animals responding^c^		
		J(0)^a^	mice	rabbits	total	mice	rabbits	total
Constrained	42	1.06–1.88	2.2 ± 0.4	1.1 ± 0.2	1.7 ± 0.3	14	4	18
Flexible	42	0.36–1.06	0.50 ± 0.15	0.45 ± 0.10	0.48 ± 0.09	3	0	3

a Range of maximum *J*(0) values defining each peptide class.

b The number of sera generating a background-corrected response greater than 0.3 OD (mice) or 0.05 OD (rabbits) to individual peptides, averaged (± SEM) over each class.

c Number of peptides in each class to which more than two sera respond.

Several lines of evidence give rise to confidence that these profiles of antigenicity are robust estimations of the intrinsic immunogenicity of MSP2. First, there is excellent agreement between the current 3D7 profile and those derived previously from experimental immunisations of mice and humans with recombinant 3D7 MSP2 [56,57]. Second, the conserved regions of MSP2 show similar patterns of antigenicity in both the FC27 and 3D7 profiles. Finally, the epitopes of an extensive panel of monoclonal antibodies to MSP2 [10,30,58,59] all coincide with peaks in the antigenicity profile (Fig. 7A).

Strikingly, both of the regions of marked conformational restriction in MSP2, the conserved N-terminal region and the region around the disulfide in the conserved C-terminal region, coincide with peaks in the experimental antigenicity profiles of both 3D7 and FC27 MSP2, and with the epitopes of several monoclonal antibodies (Fig. 7). Likewise, antigenic regions within the repeats and dimorphic regions of FC27 correspond to those that show slightly elevated low-frequency spectral densities, indicative of conformational restriction. Although the GGSA repeat, which is the most flexible region in 3D7 MSP2, shows some antigenicity, this arises from a significant response in only a single rabbit (Fig. 6), suggesting that this very flexible region is only rarely antigenic (Fig. 7). Indeed, there is a significant correlation between the antigenicity profile and relaxation-based measures of conformational flexibility: Spearman’s ρ for the comparison of the average antigenicity profiles over all mice with *J*(0) are 0.35 and 0.54 for FC27 and 3D7 MSP2, respectively, and for rabbits 0.30 and 0.21 (two-tailed p < 0.005 for all comparisons, by permutation). Thus, it appears that restricted conformational disorder within MSP2 may be a robust predictor of local antigenicity. To explore this further, we divided the peptides into two equal groups according to the maximum value of *J*(0) measured for the residues in each peptide, representing the conformationally constrained and flexible regions of MSP2 (Table 1). The peptides from constrained regions are almost four times as likely as the peptides from flexible regions to be significantly antigenic, while 85% of peptides that show significant responses in more than two animals (of either species) are from conformationally constrained regions of MSP2.

In contrast, sequence-based predictors of B-cell epitopes [60,61] perform poorly when applied to MSP2, showing weak and in some cases negative correlation with the experimental antigenicity, and failing to predict monoclonal antibody epitopes (Fig. 7). The Bepipred predictor [60] predicts 80 of 84 peptides in our array to contain B-cell epitopes, when in fact only 21 peptides reacted significantly with more than two antisera (Fig. 6), and these 21 peptides included two of the four peptides not predicted to be epitopes by this method. The approach of Kolaskar and Tongaonkar [61] performs only slightly better, predicting 35 peptides to contain epitopes, including 10 that reacted with more than two antisera.

## Discussion

Intrinsically disordered proteins are increasingly attracting interest as potential vaccine candidates against malaria [11–15,36] and other pathogens [16,17]. In spite of this, little is known about the implications of conformational disorder for the development of an effective immune response. In the case of MSP2, the recombinant protein used for both experimental immunisation and clinical trials is highly disordered, as demonstrated previously [39] and characterised further here. On the other hand, the conformation of the native GPI-anchored protein is likely to be constrained, to a greater or lesser extent, by interactions with the merozoite membrane [48] or by self-association [36,46,47,62]. These interactions also modulate the accessibility of certain epitopes on the parasite surface [10]. In light of these observations, it is evident that the efficacy of an MSP2-based vaccine is likely to depend on the appropriate targeting of epitopes that exist in an accessible form on the parasite surface. Achieving this will require an improved understanding of the way antigen conformation and flexibility modulates the specificity of the immune response. As a first step to addressing this problem, we have compared the local conformational dynamics of MSP2, as reported by ^15^N relaxation measurements, with local antigenicity as inferred from experimental animal immunisations. We find that regions of MSP2 that are most antigenic correspond to those regions in which conformational flexibility is somewhat constrained, whereas those regions that are most flexible appear to be the least antigenic.

In contrast, we find no evidence that the polymorphic regions of MSP2 are particularly antigenic. Indeed, the most polymorphic region of MSP2, the GGSA repeats of 3D7, is also the most flexible and amongst the least antigenic regions. Other polymorphic regions (green and pink in Fig. 7) are no more antigenic than are the dimorphic and conserved regions. There is evidence that the polymorphisms within these regions are selectively favoured, and although details of these selective processes are unclear, they are expected to involve host immune pressure [63,64]. As such, the lack of obvious antigenic bias towards these regions is surprising, and may highlight important immunogenic differences between recombinant MSP2 and the native parasite antigen [10].

Previous studies of structured antigens have established that increased epitope flexibility tends to increase antigenicity [65,66], in contrast to the current findings. An important distinction is that these studies have addressed epitopes that are variably flexible loops in largely structured proteins. The most flexible of these loops are unlikely to be as flexible as even the least flexible regions of MSP2. In the model structured antigen lysozyme, all residues show positive steady-state [^1^H]-^15^N NOE values greater than 0.6 [67], reflecting markedly more constrained sub-ns dynamics than is seen for any region of MSP2 (Fig 4). One possible explanation for the apparent discrepancy, therefore, may be that a moderate degree of flexibility is optimal for antigenicity, with epitopes that are either too rigid, or too flexible, being less effective. Alternatively, the determinants of antigenicity in structured and disordered proteins may differ in a more fundamental way. For example, it has been suggested that the correlation between flexibility and antigenicity observed in structured proteins reflects accessibility, rather than flexibility *per se* [68], whereas the accessibility of potential epitopes in a disordered antigen is likely to be uniformly high. Perhaps consistent with this interpretation is our observation that epitope predictors, parameterised primarily on the basis of structured antigens, perform poorly for MSP2.

The consistency of the antigenic profiles we have measured here between animals and with other previous studies in mice and in humans, strongly suggests that these profiles are determined by the intrinsic immunogenicity of the recombinant MSP2 antigen. As such, the correlation we observe between conformational restriction and antigenicity probably reflects a tendency for more flexible regions of MSP2 to be less immunogenic. The mechanistic basis underlying this tendency is currently unclear, though several possible explanations are worthy of consideration. It has been suggested that the unusual residue composition of disordered and repetitive antigens may give rise to extensively cross-reactive responses, which fail to mature into high-affinity and specific antibodies [69]. Alternatively, it may be that conformational disorder itself frustrates the process by which a mature antibody response develops. Any disordered antigen exists in a vast ensemble of distinct conformations, but a developing antibody is likely to be limited in the range of conformations it is capable of recognising. The conformational diversity of disordered antigens may therefore impose a significant barrier to antibody maturation, as proposed for the *Staphylococcus aureus* fibronectin binding protein [70]. This effect may be viewed as a conformational analogue of the epitope dilution effect recently described in the context of a polyvalent vaccine of the polymorphic antigen apical membrane antigen 1 (AMA1) [71,72]. In that context, polymorphic epitopes are ‘diluted’ relative to conserved epitopes by the combination of diverse allelic forms of AMA1, resulting in an antibody response that favours conserved epitopes. In the current context, we envisage that epitope conformations are diluted to an extent determined by the degree of disorder present in the epitope, with the result that the antibody response is biased towards more ordered epitopes.

Little is known about which MSP2 epitopes contribute to a protective immune response. Vaccine-derived protection mediated by MSP2 appears to be strain specific [23,73], suggesting that variable epitopes dominate. However this does may not be the case for the natural immune response to MSP2, where strain-specific protection has not been detected [37,74]. Nonetheless, a protective, strain-independent response is clearly desirable in the context of vaccine development. As such, our observation that conserved N- and C-terminal epitopes are amongst the most immunogenic regions of MSP2 is encouraging, although it is likely that not all of these epitopes will be accessible on the parasite surface [10].

The correlation established here begs the question of causation: is it possible to modulate the immunogenicity or antigenicity of a disordered antigen by altering its flexibility? Antigen flexibility could be modulated by directly modifying the antigen by addition of bulky residues or disulfide bonds at sites flanking a target epitope. Alternatively, simply changing the formulation of the antigen may have the desired effect. For example, the N-terminal region of MSP2 can be conformationally stabilised by interactions with lipid membranes, in a way that may better reflect the conformation of MSP2 on the merozoite surface [48]. These possibilities have important implications for the development of vaccines based on MSP2, where it is desirable to tune antigenicity towards epitopes that are conserved and exposed on the parasite surface [10,36].
